# Economic Burden of Medically Attended Respiratory Syncytial Virus Infections Among Privately Insured Children Under 5 Years of Age in the USA

**DOI:** 10.1111/irv.13347

**Published:** 2024-07-01

**Authors:** Phuong T. Tran, Sabina O. Nduaguba, Yanning Wang, Vakaramoko Diaby, Lynn Finelli, Yoonyoung Choi, Almut G. Winterstein

**Affiliations:** ^1^ Department of Pharmaceutical Outcomes and Policy, College of Pharmacy University of Florida Gainesville Florida USA; ^2^ Faculty of Pharmacy HUTECH University Ho Chi Minh City Vietnam; ^3^ Department of Pharmaceutical Systems and Policy School of Pharmacy Morgantown West Virginia USA; ^4^ West Virginia University Cancer Institute West Virginia University Morgantown West Virginia USA; ^5^ Center for Drug Evaluation and Safety University of Florida Gainesville Florida USA; ^6^ Department of Health Outcomes and Biomedical Informatics, College of Medicine University of Florida Gainesville Florida USA; ^7^ Global Value and Real‐World Evidence Otsuka America Pharmaceutical, Inc. Princeton New Jersey USA; ^8^ Center for Observational and Real‐World Evidence Merck & Co., Inc Rahway New Jersey USA; ^9^ Department of Epidemiology, College of Medicine and College of Public Health and Health Professions University of Florida Gainesville Florida USA

**Keywords:** cost, economic burden, episode of care, respiratory syncytial virus, RSV

## Abstract

**Background:**

The cost of medically attended RSV LRI (lower respiratory infection) is critical in determining the economic value of new RSV immunoprophylaxes. However, most studies have focused on intermittent RSV encounters, not the episode of care that captures the entirety of RSV illness.

**Methods:**

We created age‐ and condition‐specific cohorts of children under 5 years of age using MarketScan® data (2015–2019). We contrasted aggregating healthcare costs over RSV‐LRTI episodes to ascertaining costs based on RSV‐specific encounters only. Economic burden was estimated by multiplying costs per encounter or per episode by their respective incidence rates.

**Results:**

Average cost was higher per episode than per encounter regardless of settings (inpatient: $28,586 vs. $18,056 and outpatient/ED: $2099 vs. $407 for infants). Across ages, the economic burden was highest for infants and RSV‐LRTI requiring inpatient care, but the burden in outpatient/ED settings was disproportionately higher than costs due to higher incidence rates (for inpatient vs. outpatient episodes: $226,403 vs. $101,269; for inpatient vs. outpatient encounters: $151,878 vs. $38,819 per 1000 infant‐years). For high‐risk children, cost and burden were up to 3–10 times higher, respectively.

**Conclusions:**

With a comprehensive stratification by settings and risk condition, the encounter‐ versus episode‐based estimates provide a robust range for policymakers' economic appraisal of new RSV immunoprophylaxes.

## 1 Introduction

Respiratory syncytial virus (RSV) is young children's most common cause of lower respiratory tract infections (LRTIs) [1, 2, 3, 4, 5]. Among children under 2 years of age, RSV is responsible for 10%–35% of pneumonia cases [2, 6] and 70%–84% of bronchiolitis cases [4, 5]. Conditions putting infants at high risk for severe RSV diseases include preterm births, chronic lung disease (CLD), congenital heart disease, neuromuscular disease (ND), and immunosuppression [7]. Only supportive therapy for RSV disease is available, placing importance on immunoprophylaxis, including palivizumab, which is typically recommended only for certain high‐risk groups [8]. Additional long‐acting monoclonal antibodies and maternal vaccines have been recently approved or are in development. They are intended for prevention in broader patient groups. Thus, information on the cost of RSV medical attendance will be important for the value assessment of new preventive strategies.

Most studies that have evaluated the economic burden of RSV infection have focused on infants and hospital admission, resulting in scarce data on the economic burden for older pediatric groups and milder infections that are managed in outpatient settings [9, 10]. A systematic review on RSV costs among infants under 1 year of age published in 2022 concluded that data variability was substantial among 141 included studies and emphasized the need for nationally representative studies considering emergency and outpatient visits [9]. Finally, studies have typically focused on costs specific to single encounters with RSV diagnoses (in‐ or outpatient) rather than capturing costs for the entire episode of care (including for example follow‐up visits after hospital discharge that may not carry RSV diagnoses but are a direct consequence of the infection). Comprehensive capture of infection costs provides a more appropriate benchmark for cost‐effectiveness analyses of prevention and treatment options and delivers data for episode‐based payment models that may be considered as alternative to fee‐for‐service approaches [11, 12, 13, 14, 15, 16].

Finally, estimates of RSV burden are highly dependent on RSV infection frequency and the approach to delineate individual episodes, children's underlying comorbidities, the insurance type, and case severity [17, 18, 19, 20, 21, 22, 23, 24, 25, 26, 27]. To ensure accurate estimates of disease burden, it is therefore critical to use incidence rates and cost estimates derived from the same study population and design, considering the same units of measurement (patients, episodes, or encounters). Mismatching these units and other design features can incorrectly inflate or deflate the burden estimates.

Our previous work among children under 5 years of age estimated incidence rates of medically attended RSV infections among a national sample of privately insured patients in the USA. We stratified incidence estimates by a comprehensive set of variables such as RSV season, clinical setting (outpatient, emergency department [ED], inpatient, and intensive care unit [ICU]), age, and relevant underlying comorbidities [28]. In this study, we aimed to assess RSV‐associated costs for the same strata used in our previous work. In addition, we evaluated two approaches for calculating infection burden: (1) capture of expenditures during RSV episodes considering all costs from illness onset to the end of medical management (episode) and (2) focus on only RSV‐specific medical encounters (encounter).

## 2 Methods

### 2.1 Data Sources

The study population was established from the MarketScan® Commercial Claims and Encounters Database 2015–2019, using all enrollees with insurance coverage in noncapitated health plans. The MarketScan database includes privately insured US patients, covering more than 100 million lives over the study period. It allows longitudinal follow‐up on healthcare utilization, including inpatient and outpatient medical services and reimbursed medications dispensed in outpatient pharmacies. Enrolment detail with demographic information (e.g., age, region, and health plan type) is also available.

The University of Florida Institutional Review Board exempted this study from review because deidentified data were used.

### 2.2 Study Population and Design

Our study population included children under 5 years of age who had at least one encounter with RSV‐related lower respiratory infection (RSV‐LRI) over four consecutive RSV years (July 2015–June 2019), as described previously [28]. In addition to age‐specific cohorts of the general population of children, we developed subcohorts aligned with American Academy of Pediatrics (AAP) guidelines based on gestational age, CLD, hemodynamically significant coronary heart disease (CHD), ND, cystic fibrosis (CF), Down's syndrome, and children considered profoundly immunocompromised (IM) [8]. We estimated overall age‐specific and high‐risk stratum/age‐specific annual incidences for RSV‐LRTI using an open cohort design where children entered the study at the beginning of the year, when in ambulatory care, and when meeting age‐ and stratum‐specific inclusion criteria (e.g., when they turned 1 year of age for incidence estimates for 1‐year‐old children). Accordingly, follow‐up ended at the end of the year, when hospitalized, and when criteria for assignment to a particular stratum ended (e.g., at the second birthday).

**FIGURE 1:**
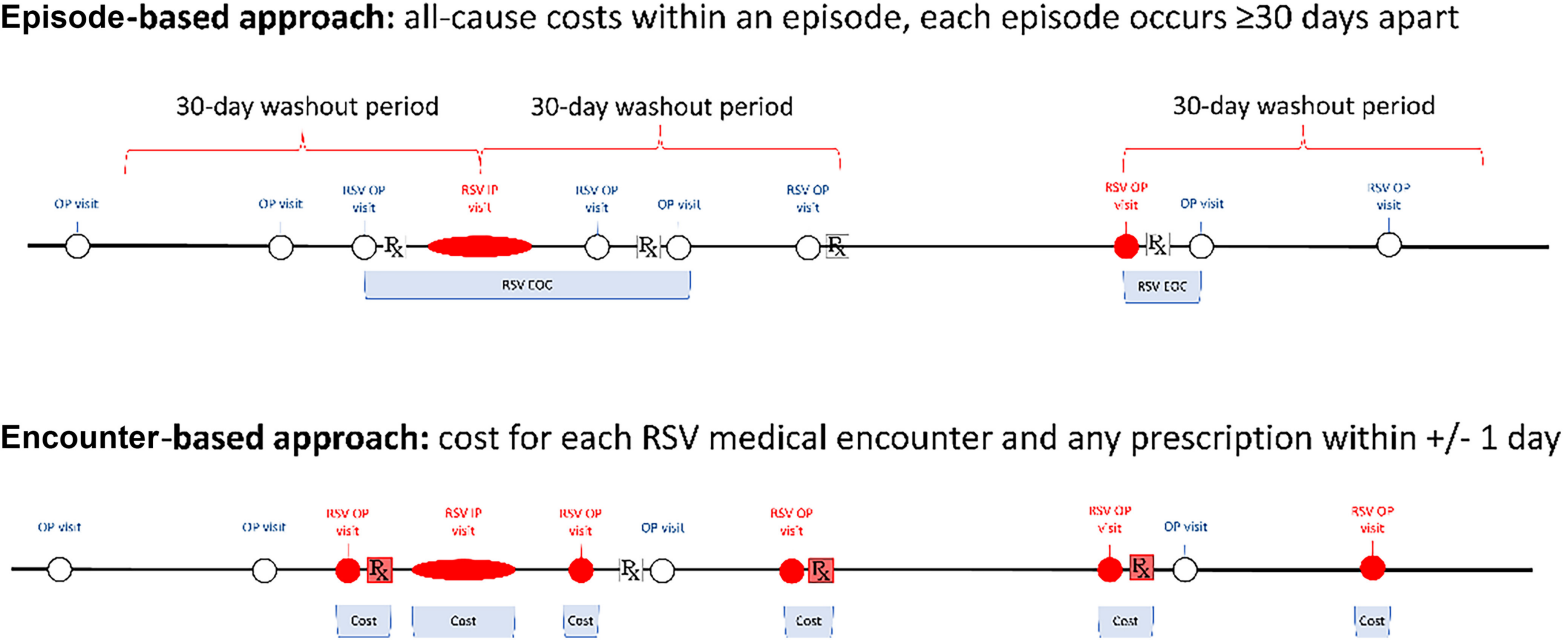
Illustration of episode‐ and encounter‐based approach to estimate total disease burden.

Consistent with our previous work [28], incidence was estimated considering either distinct episodes of an RSV‐LRTI (using a 30‐day wash‐out period between medical encounters) or counting each medical encounter with a diagnosis of RSV‐LRTI. A medical encounter with an RSV diagnosis was identified using specific RSV diagnoses (ICD‐10‐CM codes J12.1, J21.0, or J20.5), or diagnoses for bronchiolitis or pneumonia (ICD‐10‐CM codes J12, J18, J21, or J22) accompanied by an RSV confirmation code (ICD‐10‐CM codes B97.4). These two incidence measurements formed then the basis for two alternative approaches to estimate overall disease burden in this study: In the episode‐based approach, we multiplied the episode‐based incidence estimates with episode‐based costs where costs for all medical care charged within episode onset and offset were aggregated. In the encounter‐based approach, we multiplied encounter‐based incidence with encounter‐specific costs (Figure 1).

### 2.3 Cost and Burden Assessments

We reported the unit (average) cost per RSV‐LRTI episode or encounter and the total burden as the product of unit costs and incidence for a virtual cohort of 1000 children over 1 year. Costs included the amount paid by insurance and out of pocket and were upward adjusted to 2021 US dollars using the U.S. Consumer Price Index for medical care.

#### 2.3.1 Cost of RSV Episodes

We identified the RSV‐LRTI episode onset and offset based on the methodology developed in a previous study [29]. This study used a set of LRTI codes (diagnosis‐specific codes) on in‐ or outpatient encounters to identify and anchor an LRTI episode (index encounter). Diagnosis specific codes to identify index encounters that we translated into ICD‐10‐CM codes were acute bronchiolitis due to RSV (466.11, J21.0), acute bronchiolitis due to other infectious organisms (466.19, J21.1, J21.8, J21.9), acute bronchitis (466.0, J20.0‐J20.9), pneumonia due to adenovirus (480.0 J12.0), pneumonia due to RSV (480.1, J12.1), pneumonia due to parainfluenza virus (480.2, J12.2), adenovirus (079.0, B97.0), and RSV (079.6, B97.4).

**FIGURE 2:**
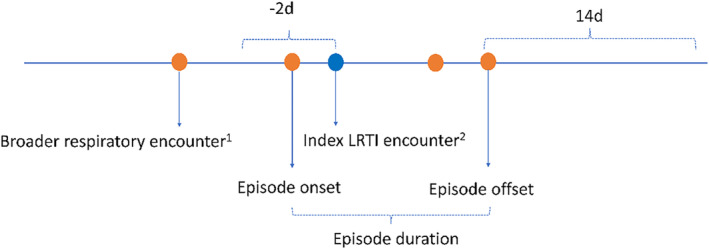
Illustration of episode duration determination. ^1^Tachypnea (786.06, R06.82), wheezing (786.07, R06.2), other respiratory distress (786.09, R06.00, R06.09), fever (780.6, 780.60, R50, R50.9, R50.81), cough (786.2, R05), acute nasopharyngitis (460, J00), acute upper respiratory infections (465, J06), acute upper respiratory infections of unspecified site (465.9, J06.9), and influenza with other respiratory manifestations (487.1, J10.1, J11.1). ^2^Acute bronchiolitis due to RSV (466.11, J21.0), acute bronchiolitis due to other infectious organisms (466.19, J21.1, J21.8, J21.9), acute bronchitis (466.0, J20.0‐J20.9), pneumonia due to adenovirus (480.0 J12.0), pneumonia due to RSV (480.1, J12.1), pneumonia due to parainfluenza virus (480.2, J12.2), adenovirus (079.0, B97.0), and RSV (079.6, B97.4). Episode onset was defined based on any encounter with relevant codes within 2 days of the index LRTI encounter. Episode offset was the first date of a LRTI‐specific or related encounter followed by at least 14 days without further LRTI‐specific or related encounters.

Determination of episode onset and offset then considered a broader set of related codes on medical encounters surrounding the index encounter including tachypnea (786.06, R06.82), wheezing (786.07, R06.2), other respiratory distress (786.09, R06.00, R06.09), fever (780.6, 780.60, R50, R50.9, R50.81), cough (786.2, R05), acute nasopharyngitis (460, J00), acute upper respiratory infections (465, J06), acute upper respiratory infections of unspecified site (465.9, J06.9), and influenza with other respiratory manifestations (487.1, J10.1, J11.1). Episode onset was defined based on any encounter with relevant codes within 2 days of the index LRTI encounter. Episode offset was the first date of a LRTI‐specific or related encounter followed by at least 14 days without further LRTI‐specific or related encounters (Figure 2).

We used a similar process to determine outpatient episode's onset and offset, except we used an outpatient (instead of inpatient) LRTI‐specific encounter as the anchor. Additionally, outpatient episodes had to be independent of an inpatient episode; that is, no inpatient encounters (for any reason) could appear between the onset and offset.

Finally, we required at least one encounter within the episode to have an RSV code to qualify as an RSV‐LRTI episode. We allowed concomitant diagnoses of other pathogens during an RSV‐LRTI episode.

To obtain cost estimates for an LRTI episode of care, for each patient, we identified the first episode in a given season year (July–June) and summed the cost of all inpatient and outpatient encounters and outpatient pharmacy dispensing within that episode.

#### 2.3.2 Costs of RSV Encounters

To determine costs for each encounter, we identified all medical encounters with RSV‐LRTI diagnosis codes and all reimbursed medications within 1 day before and 1 day after these encounters. To avoid double counting, if two RSV encounters occurred in close proximity, medication costs were only counted once (e.g., Day 1 considered prescriptions within +/− 1 day while Day 2 only considered prescriptions on Day 3). The sum of costs of all encounters (including prescription costs) was then divided by the total number of encounters to assess mean total costs and standard deviations (SDs).

All analyses were conducted using SAS 9.04.01.M6.

## 3 Results

A total of 61,121 children and 124,483 RSV‐LRTI encounters provided data. The mean estimates of episode duration were slightly longer than median estimates and were about 2–4 times longer for inpatient than for outpatient episodes (https://tabsoft.co/3QMELSV). For example, the mean and median durations for episodes that included an inpatient encounter were 8.15 (SD 7.73) and 6 days (IQR 4–10) for infants aged less than 1 year, respectively. Corresponding numbers for episodes in outpatient care were 2.84 SD (4.03) and 1 (IQR 1–3) day. Episode duration became shorter as children became older and with larger gestational age. We found the longest episode duration involving inpatient stays for infants with CHD (mean:16.51, SD 19.31 and median: 11, IQR 6–19) and shortest for children aged 4 years (mean 6.17, SD 5.39 and median: 5, IQR 3–8). Episode duration in the outpatient setting also varied across strata with a mean of less than 3 days across all age groups, and the largest mean of 6.24 (SD 6.40) for infants less than 1 year with Down's syndrome or those IM (6.75, SD 8.98). Of note, the preindex period preceding an inpatient index RSV‐LRTI was generally small (mean < 0.5 days across all strata), suggesting that in most scenarios, hospital admission followed soon after an outpatient encounter.

### 3.1 Episode‐Based Costs and Annual Burden

Mean episode costs were similar across age groups with $28,586 (SD 55,523) for episodes that included an inpatient stay for infant less than 1 year of age and $25,124 (SD 31,639) for 4‐year‐olds. The same was true for outpatient RSV‐LRTI episodes, which amounted to less than a 10th of the costs ($2099, SD 12,829 and $1848, SD 13,140 for the same age groups, Table 1). Episode costs for specific high‐risk groups were considerably higher, for example, $72,045 (SD 107,117) for infants with CLD and $94,227 (SD 219,860) for infants with congenital heart disease (Table 2, for all estimates of variation https://tabsoft.co/3QMELSV).

**TABLE 1:** Comparison of RSV‐LRTI burden by chronological age and clinical setting.

Outcome	Episode‐based				Encounter‐based				
	***N*** ^a^	Incidence (per 1000 children‐years	Mean cost (USD)	Burden (USD per 1000 children‐years)	***N*** ^a^	Incidence (per 1000 children‐years)	Mean cost (USD)		Burden (USD per 1000 children‐years)
							Medical services	Prescription drugs^b^	
**Setting**	**Inpatient**								
Age 0	4153	7.91	28,586	226,403	5258	8.44	18,056	25	151,878
Age 1	1224	1.91	24,082	46,237	1296	2.29	18,457	73	42,248
Age 2	504	0.77	25,588	18,424	682	0.97	17,154	63	16,529
Age 3	238	0.37	31,870	11,473	350	0.47	20,695	192	10,026
Age 4	96	0.16	25,124	6030	196	0.24	21,757	82	5241
**Setting**	**Outpatient**								
Age 0	43,902	48.29	2099	101,269	70,133	89.66	407	26	38,819
Age 1	20,568	20.67	1151	23,753	28,855	35.65	385	36	15,001
Age 2	8914	7.87	1121	8875	10,994	13.01	392	57	5819
Age 3	3869	3.28	1062	3442	4567	5.52	422	52	2615
Age 4	1769	1.55	1848	2882	2152	2.72	384	57	1217

^a^ Sample available to estimate costs.

^b^ Reimbursed medications dispensed in outpatient pharmacies.

**TABLE 2:** Comparison of inpatient RSV‐LRTI burden by guideline‐based high‐risk groups.

Outcome	Approach 1—Clinical episode of care				Approach 2—Medical encounter services				
	***N*** ^a^	Incidence (per 1000 children‐years)	Mean cost (USD)	Burden (USD per 1000 children‐years)	***N*** ^a^	Incidence (per 1000 children‐years)	Mean cost (USD)		Burden (USD per 1000 children‐years)
							Medical services	Prescription drug	
**Setting**	**Inpatient**								
wGA—age 0									
< 29	86	35.68	69,416	2,476,981	83	40.45	36,747	59	1,488,657
29 to < 32	94	33.15	65,950	2,186,002	105	36.16	42,925	133	1,556,963
32 to <35	384	26.13	47,774	1,248,231	508	28.73	22,742	23	654,012
35 to < 37	211	16.95	31,101	527,251	271	18.27	21,612	15	395,149
≥ 37	3378	7.62	24,169	184,145	4291	8.13	16,307	22	132,712
CLD									
Age 0	128	33.76	72,045	2,432,392	117	38.21	33,462	119	1,283,150
Age 1	Not calculated due to < 25 observations								
CHD age 0	50	36.20	94,227	3,410,577	66	55.32	23,774	89	1,319,984
IMC									
Age 0	Not calculated due to < 25 observations								
Age 1									
ND age 0	391	17.76	51,496	914,713	398	27.92	28,656	46	801,406
**Setting**	**Outpatient**								
wGA—age 0									
< 29	209	5403	6015	325,013	411	142.07	455	86	76,838
29 to < 32	343	71.25	5136	365,966	793	198.51	494	77	113,511
32 to < 35	1711	62.99	5323	335,322	3499	153.01	477	27	77,218
35 to < 37	1322	62.19	3221	200,345	2454	130.76	473	21	64,593
≥ 37	40,317	51.60	1880	96,982	62,951	93.39	399	25	39,607
CLD									
Age 0	330	71.96	5643	406,070	752	166.01	426	94	86,248
Age 1	30	70.22	11,012	773,288	100	159.78	534	29	89,871
CHD age 0	227	73.19	3690	270,062	463	197.22	521	55	113,678
IMC									
Age 0	Not calculated due to < 25 observations				123	139.57	431	63	68,873
Age 1	35	57.11	13,694	782,077	78	96.11	681	88	73,960
ND age 0	1498	32.55	4374	142,356	3019	144.20	434	38	67,997

Abbreviations: CHD, congenital heart disease; CLD, chronic lung disease; IMC, immunocompromised patients; ND, RSV‐LRTI, respiratory syncytial virus–lower respiratory tract infection; wGA, week gestational age; neuromuscular disease.

^a^ Number used to estimate costs.

In contrast to costs, the other factor determining disease burden, incidence rates, decreased substantially with increasing age, as shown in our previous work [28]. For example, the incidence rates of inpatient RSV‐LRTI were 40 times higher in infants compared to 4‐year‐old children. As a result, with an RSV‐LRTI inpatient incidence rate of 7.9 per 1000 children‐years among infants less than 1 year, the annual RSV‐LRTI burden amounted to $226,403 per 1000 infants in that age group. For 4‐year‐olds, this number had dropped to $6030 per 1000 children. Likewise, because of substantially higher outpatient RSV‐LRTI incidence rates (e.g., 7.9 inpatient vs. 48.3 outpatient per 1000 children‐years), the annual burden of RSV‐LRTI episodes in outpatient care was about half of the inpatient episode burden ($101,269 per 1000 infants less than 1 year), although individual episode costs were much smaller.

The annual burden of inpatient RSV‐LRTI among high‐risk groups was more than 10 times higher than for the overall population of infants, with an annual cost of $2,432,392 per 1000 infants with CLD and $3,410,577 with CHD (Table 2). The magnitude of these disparities was driven by both higher incidence rates and higher episode costs. Differences for outpatient RSV‐LRTI burden were less pronounced, but still about 2–4 times higher than for the general population of infants, e.g., $406,070 per 1000 infants with CLD and $270,062 with CHD.

### 3.2 Encounter‐Based Costs and Annual Burden

Incidence estimates for infections managed in the inpatient setting were similar when counting unique episodes versus encounters (e.g., 7.9 and 8.4 RSV‐LRTI hospitalization per 1000 children‐years for infants). Thus, differences in the estimates of the overall disease burden via our encounter‐based versus our episode‐based approach were driven by differences in the mean cost. Considering only the costs of the actual encounter and adjacent prescription drug claims, inpatient RSV‐LRTI encounter costs averaged about two‐thirds of the costs of an episode (e.g., $18,056, SD 27,709 for the inpatient encounter and $25, SD 213 for prescription drugs for infants, Table 1). Accordingly, disease burden estimated with the encounter‐based approach resulted in lower estimates than via the episode‐based approach (e.g., $151,878 for inpatient RSV‐LRTI vs. $226,403 per 1000 infants per year). This was also true for disease burden estimates in the outpatient setting where higher encounter‐based incidence estimates did not compensate for lower encounter‐based costs. For example, while we found almost 2 RSV‐LRTI outpatient encounters for each outpatient RSV‐LRTI episode (incidence 89.66 vs. 48.29 per 1000 infant‐years), corresponding mean encounter costs were only $406 (SD 1276) compared to $2099 (SD 12,829) for episodes, resulting in a total annual disease burden of 38,819 versus 101,296 per 1000 infants, respectively.

As with the episode‐based approach, encounter costs were similar across age groups for both inpatient and outpatient RSV‐LRTI encounters, but higher for high‐risk groups (e.g., $33,462 for inpatient encounters among infants with CLD (Table 2, for all estimates of variation https://tabsoft.co/3QMELSV). Accordingly, the disease burden for high‐risk groups was appreciably higher for both inpatient and outpatient RSV‐LRTI encounters, but costs trailed behind estimates from the episode‐based approach. In https://tabsoft.co/3QMELSV, we further present mean costs of annual estimates and their SDs in the ED and ICU settings for the encounter‐based approach. Across age groups, the costs ranged from $1526 to $3036 for ED and $44,699 to $58,200 for ICU.

The cost of prescription drugs that we considered in the encounter‐based approach was marginal (~$50 and ~$100 on average for outpatient and inpatient RSV‐LRTI encounters) compared to inpatient or outpatient medical encounter costs. The top three most frequently prescribed therapeutic classes were sympathomimetic agents (34.3%), adrenals (17.8%), and antibiotics/penicillin (17.2%) (Tables S1 and S2).

## 4 Discussion

This study of a national sample of privately insured US children provides a detailed assessment of the RSV‐LRTI burden. Incidence rates and costs were derived from the same population, allowing detailed assessments of the impact of differences in disease frequency and cost across patient groups and settings. Burden was calculated for age strata and prespecified high‐risk groups, which were identified based on AAP criteria. We also filled a previously identified gap of assessments specific to late‐term infants [9], using a mother–infant linkage and enhanced detail on gestational age in the ICD‐10‐CM era. Burden was further stratified by the type of healthcare setting in which the LRTI was managed, including assessments specific to emergency and intensive care. Finally, to our knowledge, this is also the first real‐world study contrasting the RSV‐LRTI burden estimated when derived from episodes of care versus restriction to only encounters that carried a RSV diagnosis. Three key findings of our study deserve discussion.

First, when contrasting annual disease burden estimates for inpatient RSV‐LRTI across our two approaches, we noted that the encounter‐based approach arrived at cost estimates that averaged about two‐thirds of those derived from the episode‐based approach. With very similar incidence estimates, this was attributable to differences in costs. For outpatient RSV‐LRTI, while incidence estimates were almost double for encounters when compared to episodes, the lower costs resulted in burden estimates that were less than half of estimates derived from our episode‐based approach.

Second, regardless of the approach, costs for in‐ and outpatient RSV‐LRTI were similar across age groups, but much higher for high‐risk groups than for the general population at the same age. Because RSV‐LRTI incidence varies profoundly across age groups, burden estimates showed a similarly profound decrease across ages, while the burden for high‐risk groups remained much higher due to both higher incidence and higher cost (e.g., > 10 times for inpatient RSV‐LRTI among infants with high‐risk conditions compare to the overall infant population). Considering the interplay of these two factors of age and risk conditions, burden estimates ranged from $6000 annual costs per 1000 children at age four to $3.4 million per 1000 infants with CHD.

Third, costs for management of RSV‐LRTI in outpatient settings were less than a 10th of the cost of those for inpatients. However, because incidence rates in the outpatient setting were much higher than for inpatient episodes, the annual burden of RSV‐LRTI episodes in outpatient care was about half of the inpatient episode burden. Encounter‐based estimates followed a similar pattern.

While it is expected that a sole focus on encounters with explicit RSV diagnoses will underestimate the total burden due to failure to capture healthcare services that were RSV‐related but not explicitly coded as such, consideration of all costs for all healthcare services within an episode will likely result in overestimates. Although this hypothesis is intuitive, no study has formally compared these two approaches. We therefore provided contrasts, assuming that the higher episode‐based and lower encounter‐based costs might provide helpful margins for future cost‐effectiveness studies.

Potential inaccuracies of our approaches center on the reliance on coding for RSV, which has been shown to have high specificity but lower sensitivity due to undertesting and undercoding in clinical practice [30, 31, 32]. For example, our recent modeling against national surveillance data from laboratories suggests that 40% of RSV‐LRTI might be captured in the claims data of this population [33]. We also noted that testing practice differs by children's age, high‐risk status, severity of the infection, and setting [34], which results in variable accuracy of incidence rates and, potentially, costs (if the coded and hence measured episodes in this study were not representative of the scope of healthcare services provided for all episodes). This could be the explanation for some counter‐intuitive findings regarding differences in estimated burden across age groups (i.e., higher‐risk children were more likely tested for RSV). For example, we noted that children 3 years of age had an average cost of $31,870 per inpatient RSV‐LRTI episode which was larger than that for children age two ($25,588).

Specific to our episode‐based approach, episode duration is another source of potential inaccuracy because it determines the volume of healthcare costs that are aggregated. Depending on age and risk status, we found episode durations between 5 to 17 days for inpatient RSV‐LRTIs and 2 to 10 days for outpatient RSV‐LRTIs. These durations are similar to the 50^th^ centile of LRTI symptoms reported in the literature from an average of 5 days for dyspnea to 11 days for cough [35].

Despite the limited data for comparison, our estimates are similar to a recent systematic review [10]. The average cost per inpatient RSV‐LRTI was $15,289 (95% confidence interval (CI), $14,491–$16,086) for privately insured infants under 1 year. The emergency or urgent care costs per visit were $501 ($484–$517) for privately insured infants or children under 2 years. These numbers are comparable, though slightly lower, to our encounter‐based results (adjusted to 2021 USD) of $18,056 (SD 27,709) for inpatients, $407 (SD $1379) for outpatient, and $1683 (SD 3036) for emergency visits among privately insured infants. As discussed earlier, differences can be attributable to the underlying risk (age and comorbidities) of the study population, highlighting the importance of generating risk group‐specific cost estimates.

Our study has several strengths including our fine stratification and the contrast of two approaches to determine disease burden. However, results are not generalizable to publicly insured children with commonly higher infection risk and a larger representation of those at high risk. Further research is needed to assess the disease burden in this population.

## 5 Conclusions

We compared two approaches to estimate the economic burden of RSV‐LRTI, which provide suitable margins for the lower (encounter‐based) and upper (episode‐based) bounds of the economic burden of RSV‐LRTI. We found that costs for in‐ and outpatient RSV‐LRTI were similar across age groups, but much higher for high‐risk groups than for the general population of infants and young children, yielding burden estimates 10 times the magnitude of those in the general population. Due to decreasing incidence rates by chronological age, the RSV‐LRTI burden decreased substantially as children became older. Although RSV‐LRTI costs in outpatient settings were less than a 10th of the cost of those for inpatients, higher incidence rates rendered the economic burden of outpatient episodes at about half of the inpatient episode burden.

## Author Contributions

**Phuong T. Tran:** conceptualization, investigation, funding acquisition, writing–original draft, methodology, validation, visualization, writing–review and editing, software, formal analysis, project administration, data curation. **Sabina O. Nduaguba:** conceptualization, investigation, funding acquisition, writing–original draft, methodology, validation, writing–review and editing, visualization, software, formal analysis, project administration, data curation. **Yanning Wang:** conceptualization, investigation, funding acquisition, writing–original draft, validation, visualization, writing–review and editing, formal analysis, project administration, software, data curation. **Vakaramoko Diaby:** conceptualization, investigation, funding acquisition, writing–original draft, methodology, validation, visualization, writing–review and editing, supervision. **Lynn Finelli:** investigation, funding acquisition, writing–original draft, conceptualization, methodology, validation, visualization, resources, formal analysis, project administration, writing–review and editing. **Yoonyoung Choi:** conceptualization, investigation, funding acquisition, writing–original draft, writing–review and editing, visualization, validation, methodology, software, formal analysis, data curation, supervision, resources. **Almut G. Winterstein:** investigation, funding acquisition, writing–original draft, conceptualization, methodology, validation, visualization, writing–review and editing, software, project administration, data curation, supervision, resources.

## Conflicts of Interest

Phuong Tran, Sabina Nduaguba, Vakaramoko Diaby, and Almut Winterstein received funding from Merck Sharp & Dohme LLC, a subsidiary of Merck & Co., Inc., Rahway, NJ, USA, to conduct this study. AGW has received research funding from NIH, AHRQ, PCORI, FDA, and the state of Florida and received honoraria as a consultant for Arbor Pharmaceuticals and Genentech Inc, none of which is related to this work. Vakaramoko Diaby is currently an employee at Otsuka America Pharmaceutical, Inc. Yoonyoung Choi and Lynn Finelli are employees at Merck Sharp & Dohme LLC, a subsidiary of Merck & Co., Inc., Rahway, NJ, USA.

## Supporting information


**Table S1**Frequency of top 20 therapeutic drug classes included in 1 day before and after an RSV encounter.
**Table S2**Frequency of top 40 drugs included in 1 day before and after an RSV encounter.
