# Supplementary material for: Economic Burden of Medically Attended Respiratory Syncytial Virus Infections Among Privately Insured Children Under 5 Years of Age in the USA
Source: Influenza Other Respir Viruses. 2024 Jul 1;18(7):e13347. doi: 10.1111/irv.13347 (PMC11216811; doi:10.1111/irv.13347)
Supplement: Supplementary file 1 — Table S1Frequency of top 20 therapeutic drug classes included in 1 day before and after an RSV encounter. Table S2Frequency of top 40 drugs included in 1 day before and after an RSV encounter. [file IRV-18-e13347-s001.docx]

**Supplemental table**

**Table S1. Frequency of top 20 therapeutic drug classes included in 1 day before and after an RSV encounter**

| **Rank** | **Therapeutic class** | **Count** | **Percent** |
| --- | --- | --- | --- |
| 1 | Sympathomimetic Agents, NEC | 108,078 | 34.3 |
| 2 | Adrenals & Comb, NEC | 56,001 | 17.8 |
| 3 | Antibiot, Penicillins | 54,328 | 17.2 |
| 4 | Antibiot, Cephalosporin & Rel. | 21,407 | 6.8 |
| 5 | Antibiot, Erythromycn&Macrolid | 17,874 | 5.7 |
| 6 | Antiinfect, Antibiotics EENT | 4,567 | 1.4 |
| 7 | Antihistamines & Comb, NEC | 4,454 | 1.4 |
| 8 | Repl Preps, Sodium Chlor Preps | 3,975 | 1.3 |
| 9 | Antiinf S/MM,Antifungal & Comb | 3,821 | 1.2 |
| 10 | Antivirals, NEC | 3,387 | 1.1 |
| 11 | Histamine (H2) Antagonist, NEC | 2,981 | 0.9 |
| 12 | Leukotriene Modifiers | 1,983 | 0.6 |
| 13 | Antiemetics, NEC | 1,918 | 0.6 |
| 14 | Antiinflam S/MM Agnts&Comb NEC | 1,759 | 0.6 |
| 15 | Misc Therapeutic Agents, NEC | 1,640 | 0.5 |
| 16 | Anesthetics, Local EENT, NEC | 1,493 | 0.5 |
| 17 | Gastrointestinal Drug Misc,NEC | 1,445 | 0.5 |
| 18 | Sulfonamides & Comb, NEC | 1,228 | 0.4 |
| 19 | Devices & Non-Drug Items, NEC | 1,145 | 0.4 |
| 20 | Others | 21,761 | 6.9 |

**Table S2. Frequency of top 40 drugs included in 1 day before and after an RSV encounter**

| **Rank** | **Generic name** | **Count** | **Percent** |
| --- | --- | --- | --- |
| 1 | Albuterol Sulfate | 97,040 | 30.8 |
| 2 | Amoxicillin | 40,905 | 13.0 |
| 3 | Prednisolone Sodium Phosphate | 26,684 | 8.5 |
| 4 | Cefdinir | 17,547 | 5.6 |
| 5 | Azithromycin | 17,307 | 5.5 |
| 6 | Amoxicillin/Clavulanate Potassium | 13,348 | 4.2 |
| 7 | Prednisolone | 12,271 | 3.9 |
| 8 | Budesonide | 10,812 | 3.4 |
| 9 | Levalbuterol Hydrochloride | 9,411 | 3.0 |
| 10 | Sodium Chloride | 4,021 | 1.3 |
| 11 | Nystatin | 3,236 | 1.0 |
| 12 | Brompheniramine Mal/DM Hydrobrom/PSE HCl | 3,032 | 1.0 |
| 13 | Oseltamivir Phosphate | 2,702 | 0.9 |
| 14 | Ranitidine Hydrochloride | 2,567 | 0.8 |
| 15 | Prednisone | 2,450 | 0.8 |
| 16 | Spacer, Inhalation | 2,438 | 0.8 |
| 17 | Montelukast Sodium | 1,983 | 0.6 |
| 18 | Fluticasone Propionate | 1,977 | 0.6 |
| 19 | Cefprozil | 1,651 | 0.5 |
| 20 | Antipyrine/Benzocaine | 1,486 | 0.5 |
| 21 | Sulfamethoxazole/Trimethoprim | 1,228 | 0.4 |
| 22 | Ciprofloxacin Hydrochloride/Dexamethasone | 1,045 | 0.3 |
| 23 | Cefixime | 991 | 0.3 |
| 24 | Ondansetron | 989 | 0.3 |
| 25 | Cetirizine Hydrochloride | 941 | 0.3 |
| 26 | Ibuprofen | 896 | 0.3 |
| 27 | Albuterol Sulfate/Ipratropium Bromide | 850 | 0.3 |
| 28 | Levofloxacin | 834 | 0.3 |
| 29 | Polymyxin B Sulfate/Trimethoprim Sulfate | 825 | 0.3 |
| 30 | Ipratropium Bromide | 807 | 0.3 |
| 31 | Ondansetron Hydrochloride | 799 | 0.3 |
| 32 | Beclomethasone Dipropionate | 789 | 0.3 |
| 33 | Mupirocin | 771 | 0.2 |
| 34 | Dexamethasone | 761 | 0.2 |
| 35 | Ofloxacin | 747 | 0.2 |
| 36 | Moxifloxacin Hydrochloride | 661 | 0.2 |
| 37 | Cephalexin | 617 | 0.2 |
| 38 | Lansoprazole | 609 | 0.2 |
| 39 | Triamcinolone Acetonide | 599 | 0.2 |
| 40 | Others | 26,618 | 8.4 |

**Tableau tables: RSV-LRTI costs estimated via ENCOUNTER- and EPISODE-based approaches and episode durations across children groups and clinical settings**

[https://tabsoft.co/3QMELSV](https://nam10.safelinks.protection.outlook.com/?url=https%3A%2F%2Ftabsoft.co%2F3QMELSV&data=05%7C01%7Cphuong.tran%40ufl.edu%7C9042a354e5a74205418508dbe0be7d6c%7C0d4da0f84a314d76ace60a62331e1b84%7C0%7C0%7C638350880975604506%7CUnknown%7CTWFpbGZsb3d8eyJWIjoiMC4wLjAwMDAiLCJQIjoiV2luMzIiLCJBTiI6Ik1haWwiLCJXVCI6Mn0%3D%7C3000%7C%7C%7C&sdata=OiZxOsMvLywYGPIYCdCuCFkkghVh3SFa6OV%2FDMATv6k%3D&reserved=0)

Notes: Strata without values are patient groups with number of observations smaller than 25.
